# Dimorphic Ovary Differentiation in Honeybee (*Apis mellifera*) Larvae Involves Caste-Specific Expression of Homologs of *Ark* and *Buffy* Cell Death Genes

**DOI:** 10.1371/journal.pone.0098088

**Published:** 2014-05-20

**Authors:** Rodrigo Pires Dallacqua, Márcia Maria Gentile Bitondi

**Affiliations:** 1 Setor de Biologia Geral, Universidade Federal de Mato Grosso do Sul, Campo Grande, Mato Grosso do Sul, Brazil; 2 Departamento de Biologia, Faculdade de Filosofia, Ciências e Letras de Ribeirão Preto, Universidade de São Paulo, Ribeirão Preto, São Paulo, Brazil; Baylor College of Medicine, United States of America

## Abstract

The establishment of the number of repeated structural units, the ovarioles, in the ovaries is one of the critical events that shape caste polyphenism in social insects. In early postembryonic development, honeybee (*Apis mellifera*) larvae have a pair of ovaries, each one consisting of almost two hundred ovariole primordia. While practically all these ovarioles continue developing in queen-destined larvae, they undergo massive programmed cell death (PCD) in worker-destined larvae. So as to gain insight into the molecular basis of this fundamental process in caste differentiation we used quantitative PCR (qPCR) and fluorescent *in situ* hybridization (FISH) to investigate the expression of the *Amark* and *Ambuffy* genes in the ovaries of the two honeybee castes throughout the fifth larval instar. These are the homologs of *ark* and *buffy Drosophila melanogaster* genes, respectively, involved in activating and inhibiting PCD. Caste-specific expression patterns were found during this time-window defining ovariole number. *Amark* transcript levels were increased when ovariole resorption was intensified in workers, but remained at low levels in queen ovaries. The transcripts were mainly localized at the apical end of all the worker ovarioles, but appeared in only a few queen ovarioles, thus strongly suggesting a function in mediating massive ovariolar cell death in worker larvae. *Ambuffy* was mainly expressed in the peritoneal sheath cells covering each ovariole. The levels of *Ambuffy* transcripts increased earlier in the developing ovaries of queens than in workers. Consistent with a protective role against cell death, *Ambuffy* transcripts were localized in practically all queen ovarioles, but only in few worker ovarioles. The results are indicative of a functional relationship between the expression of evolutionary conserved cell death genes and the morphological events leading to caste-specific ovary differentiation in a social insect.

## Introduction

The difference in the reproductive potential between the two female castes in *Apis mellifera* is clearly manifested in the size of their ovaries. An adult queen has larger ovaries, consisting of 160–180 ovarioles per ovary, whereas workers typically have 2 to 12 ovarioles per ovary [1]. This dimorphism becomes established by the end of larval development in response to the differential feeding regimes experienced by the larvae. Queen-destined larvae are nourished on royal jelly (a mixture of glandular secretions produced by nurse workers) throughout all five larval instars. In contrast, worker-destined larvae are fed on royal jelly up to the 3^rd^ larval instar, and then this diet is supplemented with pollen and honey [2].

Dietary components, such as the protein royalactin [3] and sugar concentration in the larval diet [4], in addition to nutrient sensing systems [5]–[7] appear to be involved in the fine-tuning of the divergent developmental trajectories. In a yet undefined manner, these signaling pathways may affect the endocrine system, thus generating the high juvenile hormone (JH) titer in queen larvae and the low titer in worker larvae [8]. The readout of this complex signaling is a differential pattern of gene expression in queen and worker castes [9]–[15].

The morphological divergence between the ovaries of the honeybee workers and queens is essential for caste specific functions, and the hemolymph JH titer has been singled out as a major factor triggering ovary dimorphism. The high JH levels in queen larvae have been shown to protect the ovaries against PCD, whereas the low titers in worker larvae are permissive to the activation of massive PCD in the ovaries [16], [17]. Although cell death has been observed in the ovaries of worker larvae as early as at the third instar, ovary morphology and size seemed to be the same in both queens and workers at this stage [18]. During the fourth instar, ovaries of queens and workers are still similar in size, histology and ultrastructure [19]. Differences in ovary size were much more evident at the fifth instar, as demonstrated by measuring the ovarian area in workers and queens during larval development [20]. Therefore, it is not clear in the literature when ovary divergence initiates, although it is generally agreed that it is intensified during the fifth larval instar.

Although PCD in the honeybee worker ovarioles has been morphologically well-characterized and related to nutritional status and JH titers, little is known about the genes involved in caste-specific ovary differentiation. The majority of the studies on the molecular biology of caste differentiation have used RNA obtained from worker and queen whole body samples [9]–[15]. As far as we know, only a few studies [7], [21], [22], [23] have focused on genes expressed in the larval ovaries of queens and workers. Using Representational Difference Analysis, Humann and Hartfelder [22] found ESTs representing homologs of known genes and also several unpredicted genes, including two putative long noncoding RNAs that mapped to a previously identified quantitative trait locus for ovariole number variation in the honeybee [24]. However, none of the known cell death genes were found in this differential gene expression screen.

As the core machinery of PCD is highly conserved throughout evolution [25], we searched the *A. mellifera* genome for candidate genes using known *Drosophila* cell death genes as queries. We searched for genes that could be involved in apoptotic as well as in autophagic cell death, since these have been reported to act synergistically in many tissues [26], [27]. Several components of the cell death machinery have been identified and are well-characterized in *D. melanogaster*, including the Apaf-related killer gene (*ark*) [28], displaying orthology relationship with a gene encoding the mammalian Apoptotic peptidase activating factor 1 (*Apaf-1*), as well as genes encoding the pro- and anti-apoptotic B-cell lymphoma 2 (Bcl-2) family members [29], [30].

In mammals, Apaf-1 is the adapter molecule that requires cytochrome c for caspase activation, and the release of cytochrome c from mitochondria is controlled by members of the Bcl-2 family. Upon binding to cytochrome *c*, Apaf-1 forms a multi-molecular complex including pro-caspase and ATP, known as apoptosome, which activates an initiator caspase thus triggering the dismantling of intracellular components, including cleavage of target proteins, DNA fragmentation and membrane blebbing, among other events [31]–[33].

The Apaf-1 homolog, Ark, is required for cell death activation in *Drosophila* [34]. Upon apoptotic signals, the apical caspase DRONC coassembles with Ark and cytochrome c into a large apoptosome complex to trigger cell death [35].

Although Bcl-2 proteins do not appear to play a critical role in the release of mitochondrial factors, such as cytochrome c, for apoptosis induction in *Drosophila*, these proteins are found in mitochondria and there is growing evidence that they are central regulators of apoptosis also in *Drosophila* [36].

Here we identified homologs of the pro-apoptotic *ark* gene and the anti-apoptotic Bcl-2 family member in *Drosophila*, *buffy*. Transcript levels of the honeybee genes, namely *Amark* and *Ambuffy*, were quantified and localized in queen and worker ovaries throughout the fifth larval instar when caste-specific ovary dimorphism takes place. Our results suggest that a balance between the expression of both *Amark* and *Ambuffy* genes is important for the regulation of ovariole death/survival, thus influencing the reproductive potential of *A. mellifera* queens and workers.

## Results

### Identification and Structural Analysis of *Amark* and *Ambuffy* Genes

**Figure 1:**
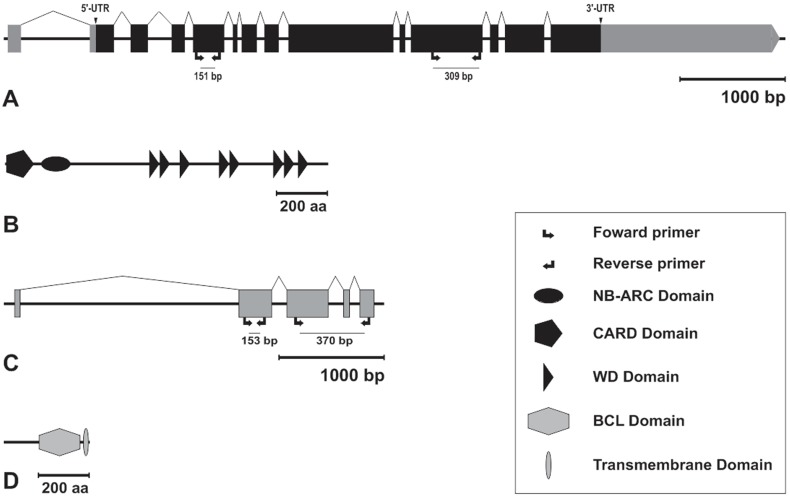
Gene and protein architectures. Schematic representations of *Amark* (A) and *Ambuffy* (C) gene sequences and their respective predicted proteins, Amark (B) and Ambuffy (D). Exons were manually annotated to the corresponding genomic scaffold using Artemis 7.0 tools or automatically annotated in BeeBase website (http://www.hymenopteragenome.org/beebase/?q=home). The 5′-UTR and 3′-UTR (gray) regions are indicated for the *Amark* gene. Protein domains were predicted using bioinformatics tools from SMART and the NCBI conserved domain database. Scale (bars on the right) indicate size of the genomic sequences (bp: base pairs) and protein sequences (aa: amino acids). Arrows show the position of the primers used for *Amark* and *Ambuffy* transcript quantification by qPCR (left) and localization by FISH (right).

The *Amark* (GB52453-GenBank accession number XR_120278.1) coding sequence comprises 3,990 nucleotides distributed in 13 exons (Figure 1A). The sequence was mapped in the genome scaffold Group 2.17 and its predicted protein showed the following evolutionary conserved domains: an N-terminal Caspase Activation and Recruitment Domain (CARD), an NB-ARC domain (Nucleotide-Binding adaptor) and C-terminal WD-40 repeat domains (Figure 1B). The search for Bcl-2 family orthologs returned only one inhibitor of apoptosis candidate, here called *Ambuffy* (GB49154 - GenBank accession number XM_395083.4). Its coding sequence is composed of 948 nucleotides distributed in 5 exons mapping in the genome scaffold Group3.14 (Figure 1C). The predicted protein has a typical Bcl domain near the C-terminus, and further downstream of it a transmembrane domain (Figure 1D).

### Caste-specific Expression of *Amark* and *Ambuffy*

**Figure 2:**
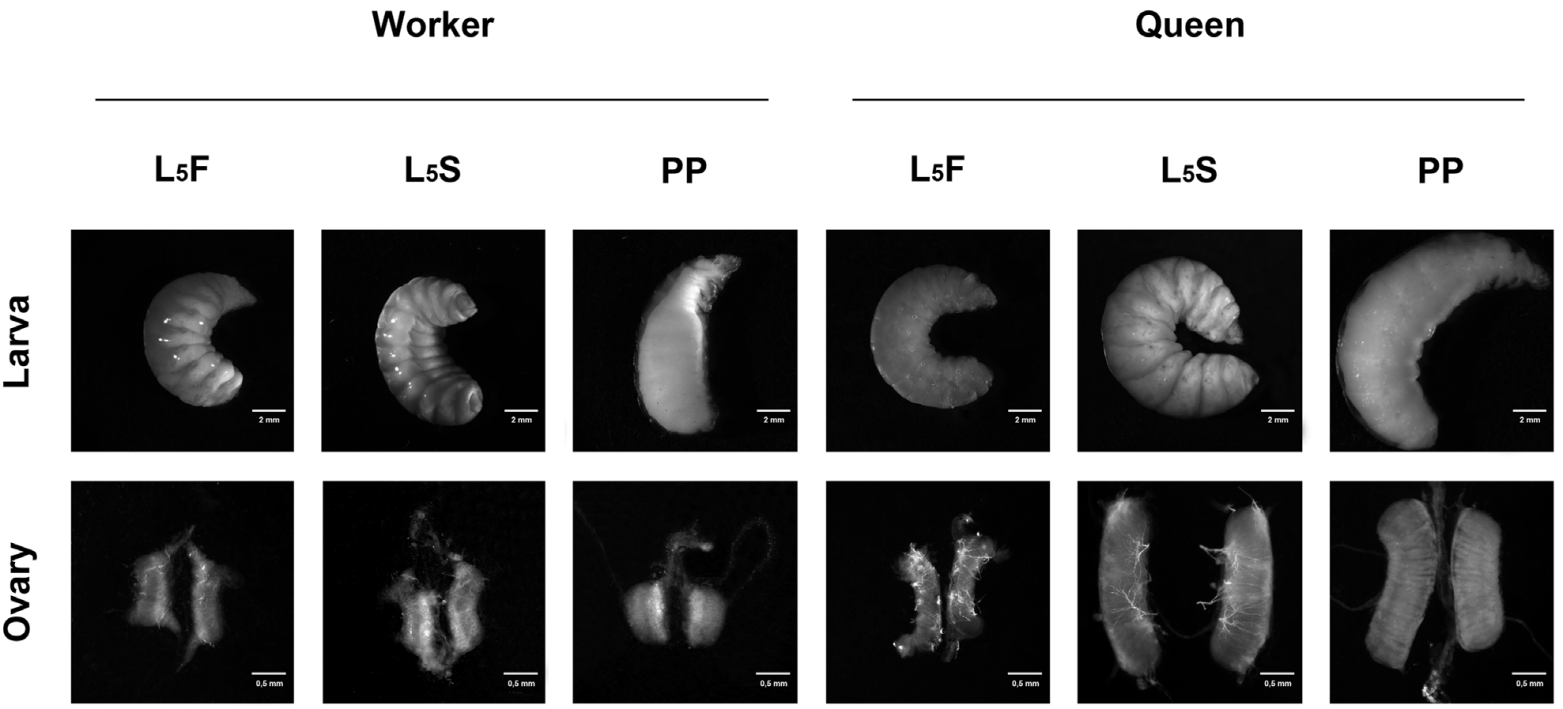
Honeybee developmental stages. Developmental phases and ovaries of honeybee workers and queens in the fifth larval instar, which is subdivided into feeding (L_5_F), cocoon-spinning (L_5_S) and prepupal (PP) phases.

**Figure 3:**
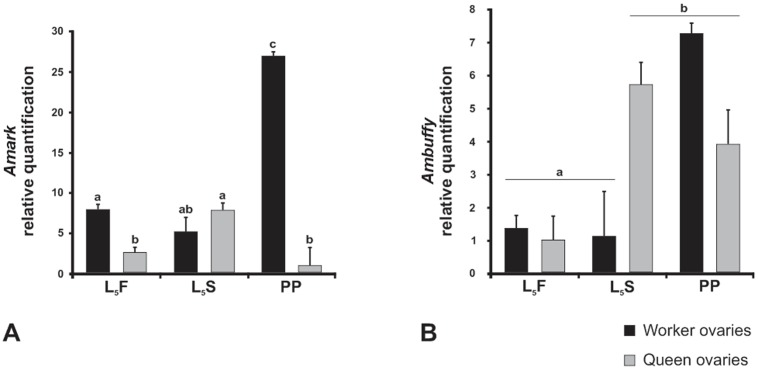
Gene expression profiles in honeybee ovaries. Relative quantification (RT-qPCR) of *Amark* (A) and *Ambuffy* (B) transcripts in the ovaries of queens and workers in the feeding (L_5_F), cocoon-spinning (L_5_S) and prepupal (PP) phases of the fifth larval instar. The gene encoding an *A. mellifera* ribosomal protein (*Amrp49*) was used for normalization. Each column represents the mean of three independent samples, each composed of five ovary pairs. Different letters indicate significant differences between groups (p≤0.001).

*Amark* and *Ambuffy* transcript levels were quantified in the ovaries of queens and workers at the time points of the fifth larval instar specified in Figure 2. The two genes were expressed in the ovaries of both castes throughout the entire fifth larval instar (Figure 3), although at different levels.

*Amark* expression showed a threefold increase in the ovaries of worker larvae at the later phase of the fifth instar (PP phase). A minor increase in transcript levels was detected in queens from the L_5_F to the L_5_S phases, but this was followed by decay to basal levels at the PP phase. Importantly, striking differences in the levels of *Amark* transcripts between the castes were evident at the PP phase when workers showed more than 25 fold transcripts than queens (Figure 3A). This is consistent with a presumed role of *Amark* as a cell death activator for ovariole degeneration in worker larvae.

*Ambuffy,* a putative cell death inhibitor, showed an increasing expression in the ovaries of worker and queen larvae. However, *Ambuffy* transcript levels increased earlier in queen ovaries (at the L_5_S phase) than in worker ovaries that showed increased transcript levels only in the later PP phase (Figure 3B).

Therefore, notable differences were found in *Amark* and *Ambuffy* transcript profiles in the developing ovaries of workers and queens.

### Spatial Localization of *Amark* and *Ambuffy* Transcripts

#### *Amark*

**Figure 4:**
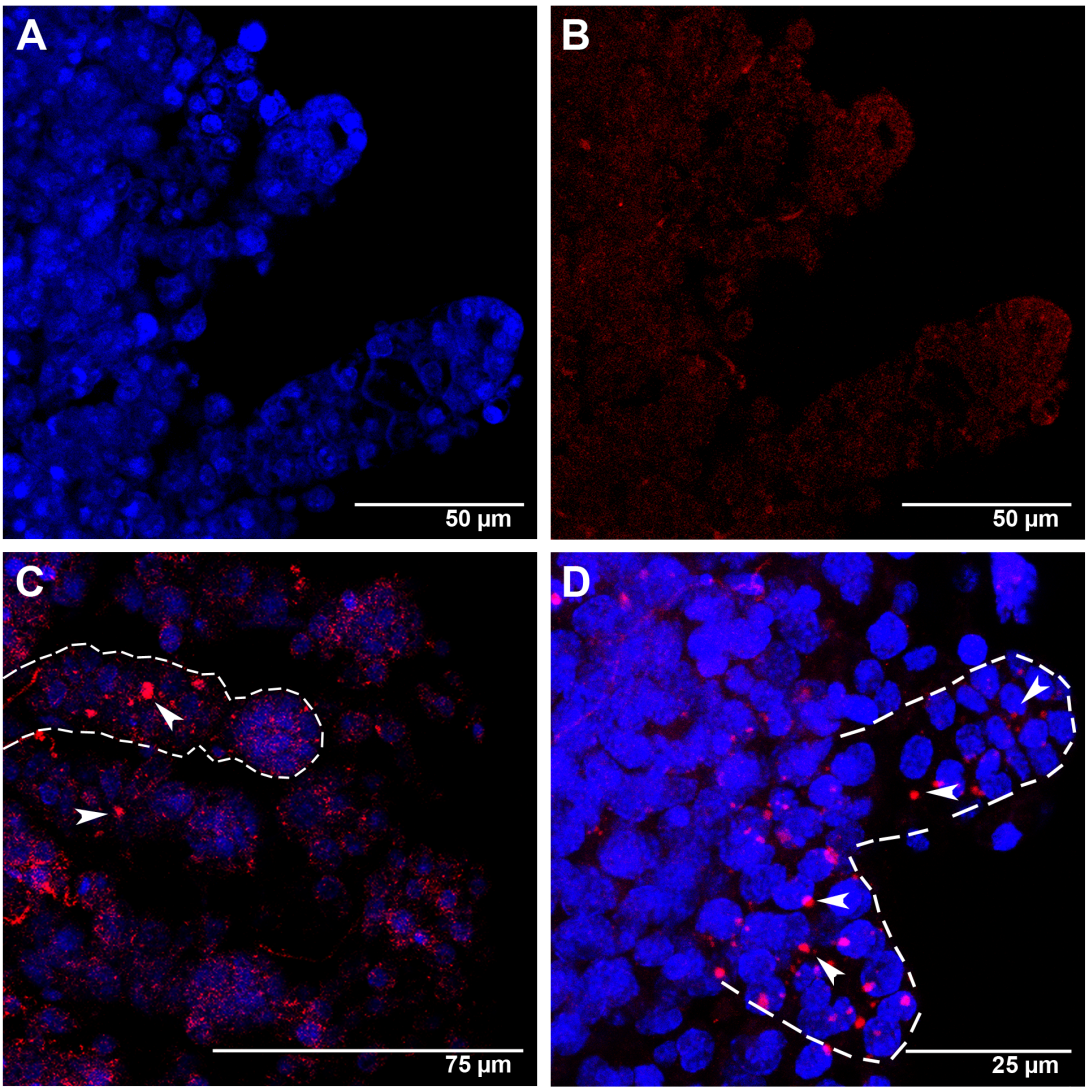
*Amark* transcript localization in worker ovaries at the L_5_F phase of the fifth larval instar. (A) Ovarioles showing DAPI-stained nuclei. (B) The same ovarioles as in A, but labeled with the AlexaFluor555-*Amark* sense probe (FISH negative control), shows only a reddish background coloration. (C and D) Ovarioles labeled with the AlexaFluor555-*Amark* antisense probe and DAPI: the dashed line in C highlights an ovariole with large *Amark* foci (red) in the intermediary region (arrowheads). *Amark* foci (arrowheads in D) are also concentrated at the apical region of some ovarioles (shown in higher magnification and outlined by dashed lines in D).

**Figure 5:**
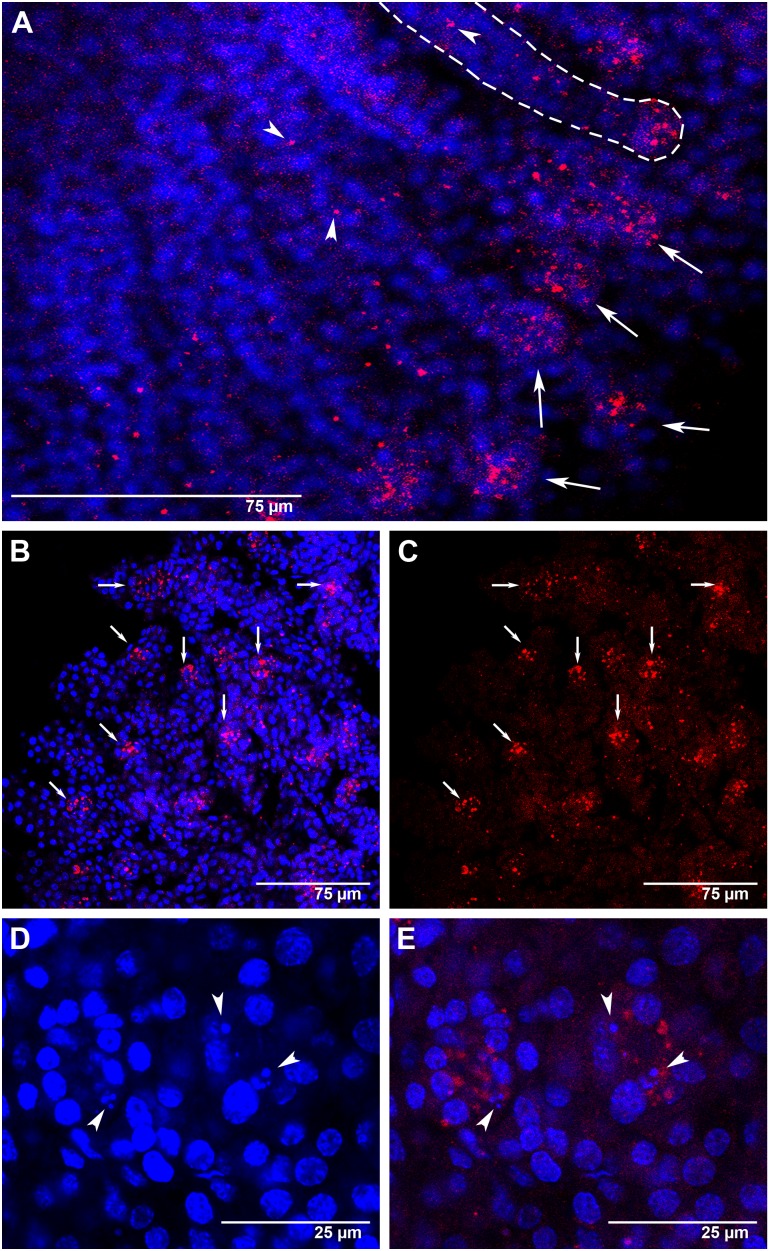
*Amark* transcript localization in worker ovaries at the L_5_S and PP phases of the fifth larval instar. FISH with AlexaFluor555-labeled *Amark* antisense probe (red foci). Cell nuclei stained with DAPI (blue). (A) An L_5_S-phase ovary showing *Amark* transcripts highly concentrated at the apical end of the ovarioles (arrows). *Amark* foci are also seen outside the apical region (arrowheads). This pattern of *Amark* labeling is generalized throughout the worker ovaries. (B and C) At the end of the fifth larval instar (PP phase) the ovary continues to show *Amark* transcripts concentrated at the apical end of the ovarioles (arrows). (D) Detail showing small-sized degenerating nuclei (arrowheads) at the tip of the ovarioles (PP phase). (E) The same ovary as seen in D, but showing *Amark* foci (arrowheads) in the region where degenerating nuclei were identified.

**Figure 6:**
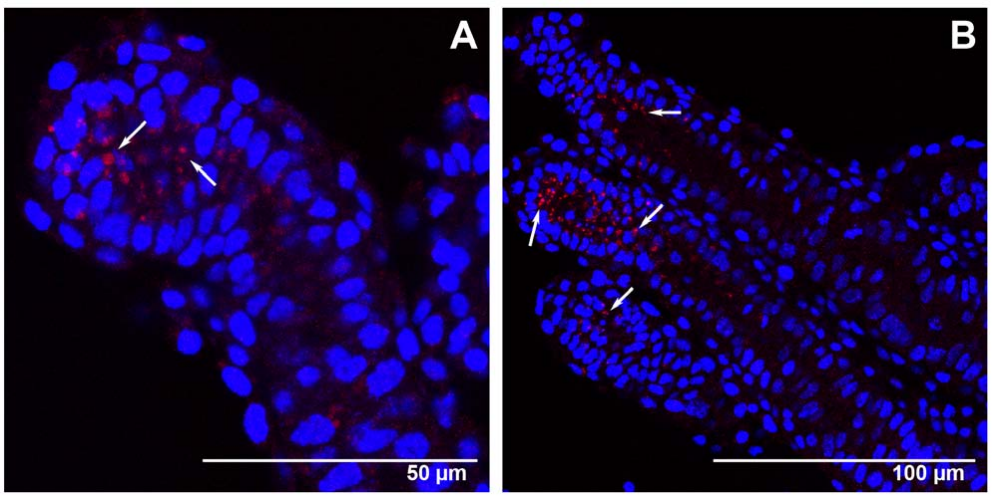
*Amark* transcripts localization in the ovaries of queens at the PP phase of the fifth larval instar. FISH with AlexaFluor555-labeled *Amark* antisense probe (red foci). Cell nuclei stained with DAPI (blue). (A and B) Details of the few ovarioles showing *Amark* foci (arrows).

In agreement with the RT-qPCR data, *Amark* transcripts were localized in the larval ovaries of workers (Figures 4, 5) and queens (Figure 6). Figures 4A–D shows ovaries of L_5_F-phase workers. Figure 4A is a DAPI-stained ovary highlighting cell nuclei and cell distribution. The same ovary, but incubated with *Amark* sense probe as a negative control is shown in Figure 4B. Labeling with the antisense probe evidenced *Amark* foci in the cytoplasm of the ovariole cells (Figures 4C, D). Well-defined foci were seen in the intermediary region of the ovarioles (which contain the presumptive, still undifferentiated, germline and somatic cells) (Figure 4C), and also in the apical region (Figure 4D).

*Amark* foci were always detected in the cytoplasm. Particularly in Figure 4D, the position of some foci may suggest the presence of *Amark* transcripts in cell nuclei. However, this figure is an image reconstruction generated by superimposing eleven successive optical sections (approximately 0.5 µm of distance between sections). The analysis of the individual images captured from different angles in high magnification (data not shown) ensures that all foci are localized in the cytoplasm.

In workers at the subsequent L_5_S phase, *Amark* foci were mainly localized at the apex of each ovariole (Figure 5A). The intermediary region of these ovarioles also showed *Amark* foci, although in a lesser amount and sparsely distributed (Figure 5A). *Amark* foci remained concentrated at the apices of the ovarioles of workers at the PP phase (Figure 5B, C). This spatial distribution is consistent with the occurrence of extensive programmed cell death at the apices of the ovarioles at the end of the fifth larval instar. In these ovarioles we also observed that *Amark* foci frequently co-localize with DAPI-stained small-sized nuclei apparently undergoing degradation (Figure 5D, E). Such nuclei were evident in all confocal optical planes, thus ensuring that they do not represent tangential sections. Co-localization of small-sized nuclei and *Amark* foci lends further support to the hypothesis of a role for this gene in ovariolar cell death.

In contrast to what was observed in the ovaries of workers at the end of the larval stage (PP phase), *Amark* foci were localized in a few queen ovarioles, and mainly so in the apical region (Figure 6A, B).

The FISH results showed a generalized presence of *Amark* foci in the apical ends of the worker ovarioles, whereas they were restricted to a few queen ovarioles. *Amark* foci abundance and spatial distribution in the ovaries are consistent with a caste-specific role of this gene in ovariole resorption.

#### *Ambuffy*

**Figure 7:**
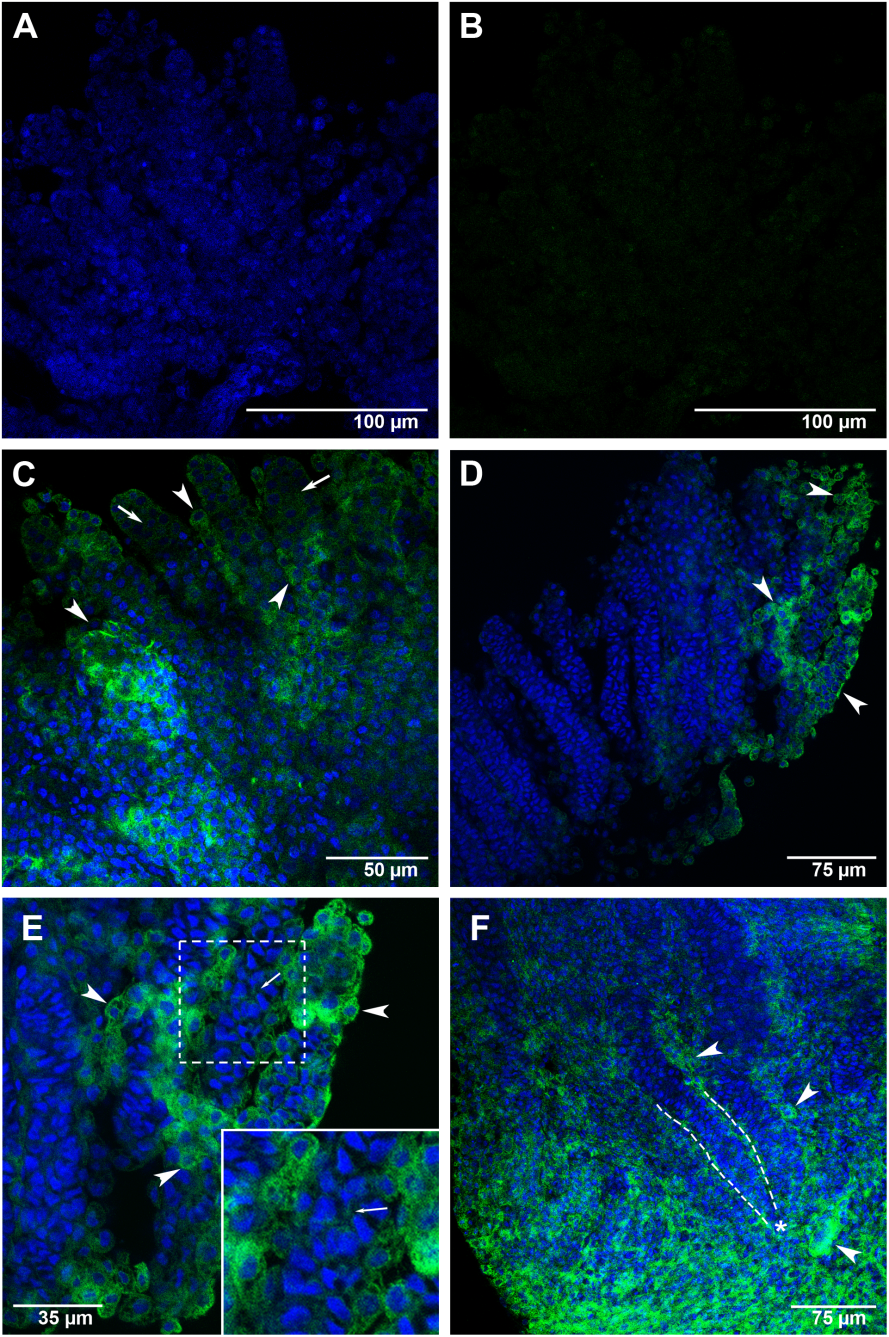
*Ambuffy* transcripts localization in the ovaries of workers at the L_5_F, L_5_S and PP phases of the fifth larval instar. (A) An L_5_F-phase ovary incubated with DAPI for cell-nuclei staining (blue). (B) The same ovary labeled with the AlexaFluor488-*Ambuffy* sense probe (negative control) shows a greenish background coloration, but not *Ambuffy* foci. (C–F) Ovaries incubated with the AlexaFluor488-*Ambuffy* antisense probe (green foci) and DAPI. Arrowheads and arrows point to peritoneal sheath cells and ovariole cells, respectively. (C) An L_5_F-phase ovary showing *Ambuffy* foci mainly in the peritoneal sheath cells involving the ovarioles. (D) An L_5_S-phase ovary showing *Ambuffy* foci in the peritoneal sheath cells of a few ovarioles (seen at the right of the figure). The remaining ovarioles showed weak, or did not show, *Ambuffy* foci. (E) Detail of the ovary seen in D, highlighting the high concentration of *Ambuffy* foci mainly in the peritoneal sheath cells, but also in the ovariole cells, as shown in higher magnification. (F) A PP-phase ovary showing high concentration of *Ambuffy* foci in its basal region. The asterisk marks the basal stalk of an ovariole (evidenced by dashed lines).

**Figure 8:**
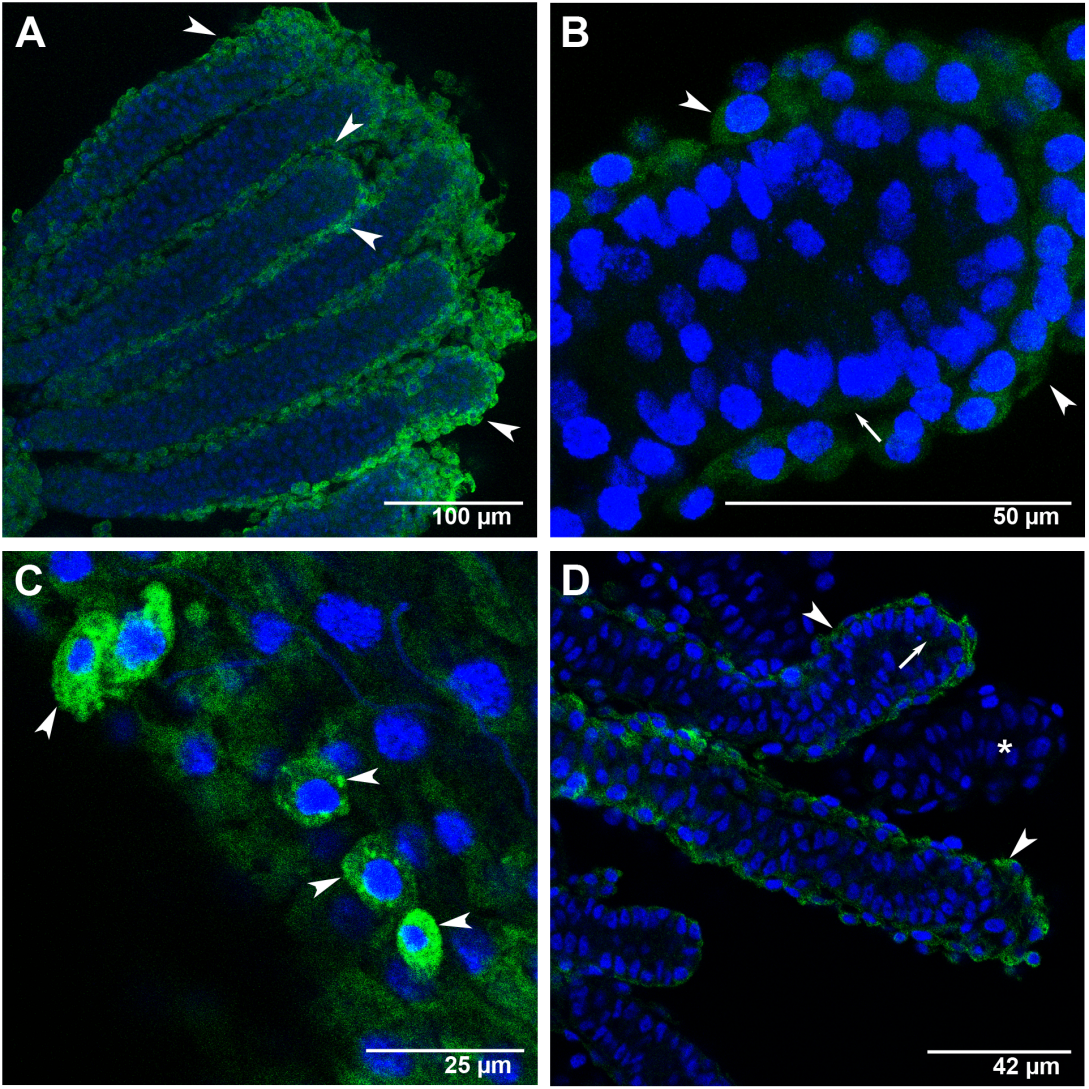
*Ambuffy* transcripts localization in the ovaries of queens at the L_5_F, L_5_S and PP phases of the fifth larval instar. FISH with AlexaFluor488-labeled *Ambuffy* antisense probe (green foci). Cell nuclei stained with DAPI (blue). Arrowheads and arrows point to peritoneal sheath cells and ovariole cells, respectively. (A) An L_5_S-phase ovary showing *Ambuffy* foci in the cytoplasm of the peritoneal sheath cells covering all ovarioles. (B) Apical portion of one of the ovarioles shown in A: *Ambuffy* foci were evident in the peritoneal sheath cells, but barely seen in the interior of the ovariole. (C) Detail of a L_5_F-phase ovariole in higher magnification: *Ambuffy* expression is clearly higher in the peritoneal sheath cells than in the ovariole cells. (D) At the PP phase, *Ambuffy*-labeling was comparatively less intense than in the previous L_5_S phase shown in A. The asterisk marks an ovariole where *Ambuffy* foci were no longer evident.

Regarding the localization of *Ambuffy,* foci were mainly detected in the cytoplasm of the peritoneal sheath cells that involve each ovariole. This was seen both in workers (Figure 7) and in queens (Figure 8), but with caste-specific differences concerning foci distribution in the ovaries and intensity.

Figure 7A shows an L_5_F-phase worker ovary stained with DAPI to highlight the ovarioles. In Figure 7B, the same ovary is shown after incubation with *Ambuffy* sense probe as a FISH negative control. Figures 7C–F show worker ovaries incubated with the *Ambuffy* antisense probe and DAPI. The transcript probe signal in L_5_F worker ovaries (Figure 7C) was more intense in the peritoneal sheath cells than in cells inside the ovarioles. At the next developmental phase, L_5_S, *Ambuffy* foci were mainly concentrated in the peritoneal sheath cells of a few ovarioles (Figures 7D, E). At the PP phase, *Ambuffy* transcripts signals showed great intensity at the basal stalk region of the ovarioles (Figure 7F), and at the peritoneal sheath cells surrounding each remaining ovariole.

In queens at the L_5_S phase, *Ambuffy* transcripts were detected in all the examined ovarioles. Transcripts predominated in the peritoneal sheath cells (Figure 8A) and stained foci were barely seen in the ovariole inner cells (Figure 8B). Figure 8C clearly shows some peritoneal sheath cells expressing *Ambuffy* in an ovariole of a queen at the L_5_F phase. A similar pattern of *Ambuffy* transcript labeling was also observed at the PP phase, but labeling intensity was weaker in comparison to that seen in the previous L_5_F and L_5_S phases (Figure 8D).

The distribution patterns of *Ambuffy* foci in the ovaries of workers and queens are consistent with the view that the expression of this gene is important to protect ovarioles from activating the PCD machinery.

## Discussion

Studies concerning PCD in insect ovaries have mainly focused on the oogenesis in *D. melanogaster*, where PCD has been linked to the resorption of abnormally developed ovarian follicles. In this case, apoptotic and autophagic machinery may act synergistically through distinct genetic pathways to execute cell death [37]–[39]. *A. mellifera* represents an interesting model organism to investigate this process, since the naturally occurring ovary dimorphism in worker and queen castes involves both autophagic and apoptotic cell death [40]. To our knowledge, there are no data on the expression of cell death genes in the differentiating ovaries of social insect castes. A recent study using global gene expression analyses [15] found twelve differentially expressed PCD genes at specific time-points of ovary dimorphism establishment between honeybee castes. Consistent with this, the majority of these PCD genes showed higher expression in worker than in queen larvae. However, these results were obtained from RNA samples of whole larval body extracts, thus making difficult to establish a connection between the general expression of these genes and the massive death of ovarioles in worker-destined larvae.

We herein used the ovaries of honeybee workers and queens to investigate the expression of two evolutionarily conserved genes involved in PCD. The expression of *ark* and *buffy* homologs in *A. mellifera*, here named *Amark* and *Ambuffy*, was investigated in the ovaries throughout the fifth larval instar when cell death is a prominent feature leading to the dimorphic phenotypes. As commented above, differences in ovary morphology and size between worker and queen castes were clearly evident at the fifth instar [18], [19], [20]. Similarly, higher levels of *Amark* transcripts were detected in the ovaries of workers (but not in queens) at the fifth instar than at the fourth instar, as we could verify using semiquantitative RT-PCR (**Figure S1**). Together, such information guided our decision of choosing the fifth instar for our FISH analysis of cell death gene expression in the ovaries of the honeybee castes.

RT-qPCR data and FISH images allowed us to establish a relationship between *Amark* and *Ambuffy* expression profiles and transcript localization in the developing ovaries of workers and queens.

### *Amark,* a Potential Pro-apoptotic Gene in the Larval Ovaries

In *Drosophila*, Ark is necessary for apoptosome formation and apoptosis induction. The *Aedes aegypti* ortholog of *ark* was identified based on sequence similarity [41]. The same strategy was used by us to search *ark* gene in the honeybee genome. This returned only one high score match (Amark) with the three typical domains: a death fold domain (CARD) [42]–[44], an NB-ARC domain that regulates protein-protein interactions during cell death [45], and WD 40 domains that in the *Drosophila ark* gene are involved in apoptosis induction in the presence of death signals [44]. Therefore, a role of *Amark* in cell death activation can be inferred by sequence similarity, and can be further proven by investigating its functionality in apoptosome formation.

Apoptotic and autophagic cell death pathways are not totally independent, as genes involved in one pathway may also be regulated in the other, suggesting that both share common molecular components [46]. In *Drosophila*, the involvement of *ark* in autophagic cell death in the larval salivary glands has already been suggested [46]–[48]. In addition, cell death failed to occur in the larval salivary glands of *ark*-deficient mutant flies [49]. Whether *Amark* is exclusively involved in apoptosis in the honeybee ovaries, or also has a role in autophagic cell death is unknown, although both PCD types have been morphologically identified during ovariole resorption in worker larvae [40].

*Amark* expression, as quantified by RT-qPCR, was significantly upregulated in the ovaries of workers during the last phase of the fifth larval instar (PP phase), coinciding with resorption of most of the ovariole primordia [16], [17]. Consistent with the maintenance of ovariole primordia integrity in queens, there was no such increase in *Amark* expression in queen ovaries.

In PP phase workers, disintegrating cell nuclei and *Amark* transcript foci were primarily located and concentrated in the apices of the ovarioles. This pattern of *Amark* foci localization was observed in the large majority of the worker ovarioles. Interestingly, disintegrating fusomes (a germline-specific organelle containing the cytoskeletal proteins actin and spectrin) were observed at the apices of larval ovarioles [17], and fusome disintegration has been seen as a major morphological signal of PCD in the ovaries. It is, however, not clear whether this represents a cause-and-effect relationship or a mere coincidence.

A previous study [16] showed TUNEL reaction-labeled apoptotic cells in the ovary midline of L_5_F worker larvae; the apical cells exhibited little evidence for apoptosis at this and at the next L_5_S developmental phase. The interpretation was that the degeneration of ovariole primordia starts in the midline region of the ovarioles, which contains the germline cells [19], [40], and subsequently extends to the other regions of the ovarioles. In support to this view, we found *Amark* labeling in the intermediary region of the ovarioles of workers at the L_5_F phase, and at the subsequent L_5_S phase, *Amark* foci were seen mainly at the apical cells. *In situ* labeling by TUNEL identifies DNA fragmentation resulting from apoptotic signaling cascades. As an apoptosis inducer, however, the action of *Amark* precedes DNA fragmentation and is needed at the beginning of the apoptotic process, prior to caspase activation and cellular substrates degradation. Supposedly, *Amark* is expressed in cells that later should be TUNEL-positive. Importantly, *Amark* expression data, as well as TUNEL-labeling experiments [16] are consistent with a temporally organized cell death program along the ovariole apical-basal axis.

*Amark* foci were also identified in queen ovaries. Although cell death has been demonstrated as predominantly occurring in worker ovary, it is not limited to this caste, since clear signs of cell degeneration have also been observed in queen ovaries [19]. Therefore, the higher levels of *Amark* transcripts in the ovaries of PP phase-workers compared to queens at the same phase, and the localized expression in the apices of most worker ovarioles, but in only few of the queen ovarioles, make this gene a strong candidate for participating in the process of caste-specific ovary dimorphism determination.

### *Ambuffy,* a Potential Anti-apoptotic Gene in the Larval Ovaries

Proteins of the evolutionarily conserved Bcl-2 family are regulators of apoptosis [50]. Complex interactions between members of the Bcl-2 family regulate cell death/viability in mammals and in *Caenorhabditis elegans* [51]. Members of the Bcl-2 family have been classified according to their roles as anti- or pro-apoptotic proteins. Such opposite roles depend on the presence of the conserved Bcl-2 homology domains, BH1, BH2, BH3 and BH4. Anti-apoptotic Bcl-2-related proteins share sequence homology to the core Bcl-2 family members particularly within the four homology domains, whereas pro-apoptotic family members in general lack the N-terminal BH4 domain, or share sequence homology with the Bcl-2 protein family only through the BH3 domain [50], [51]. Containing the four BH domains and a C-terminal transmembrane domain, Ambuffy is, thus, a *bona fide* anti-apoptotic Bcl-2 protein, as proteins in this class contain the four BH domains, with the BH4 domain being critical for the anti-apoptotic activity [52].

The *A. mellifera* genome has just one gene encoding a Bcl-2 family member that shares 46% similarity to Buffy, one of the two Bcl-2 proteins of *D. melanogaster* [29], [30]. The other fruit fly Bcl-2 protein, Debcl, shows 39% similarity with Ambuffy. Like *D. melanogaster*, the *Bombyx mori* genome also has two *bcl-2* genes, one of them, *Bmbuffy*, encodes a protein that share 51% similarity with *D. melanogaster* Buffy [53] and 26% similarity with Ambuffy, as revealed by BLASTP analysis. *Tribolium castaneum* genome also has a single Bcl-2-like sequence (GenBank accession number XM_961548), which shares 40% similarity with Buffy and Ambuffy sequences.

The temporal dynamics of *Ambuffy* expression variation, as revealed by RT-qPCR transcript quantification, were clearly caste-specific. The six fold increase in *Ambuffy* transcript levels in the ovaries of queens at the intermediary (L_5_S) phase of the fifth larval instar, and the maintenance of a similar high level of transcripts at the PP phase, is consistent with a function in protecting ovarioles against cell death and degeneration. In the ovaries of workers, increase in *Ambuffy* transcripts levels was only seen at the final PP phase, perhaps for protection of the surviving ovarioles. These results were compared with the FISH-images localizing *Ambuffy* transcripts in the ovaries of queens and workers. *Ambuffy* labeling was mainly localized in the epithelial peritoneal sheath covering each ovariole and separating this from one another. Although in lesser amounts, transcripts were also found within the ovarioles. Such localized expression was verified both in queens and in worker ovaries, but with notable differences. During the L_5_S phase, *Ambuffy* labeling was seen in all queen ovarioles, whereas in workers, only a small number of ovarioles showed *Ambuffy* foci. Like the developmental dynamics of *Ambuffy* transcript levels, the FISH-labeling patterns would be consistent with a role for *Ambuffy* in protecting all the ovarioles of queen-destined larvae from cell death, as well as those ovarioles that will survive in the worker-destined larvae. This hypothesis receives support from experiments performed in *Drosophila,* where the activity of Buffy in cell death inhibition has been demonstrated [30]. Furthermore, consistent with the presence of *Ambuffy* transcripts in the ovaries, an immunohistochemistry assay using honeybee workers at the third and fourth larval instars and a commercially available antibody against mammalian Bcl-2 detected the target protein in the larval ovaries [54]. Together, these findings are consistent with a role for *Ambuffy* as an anti-apoptotic gene in the larval ovaries of the honeybee.

Interestingly, in the ovaries of PP-phase workers *Ambuffy* foci were also concentrated at the basal stalk region, which differentiates at the L_5_S phase and is temporarily preserved from cell death in spite of the extensive degeneration of ovariole primordia [19]. This is consistent with *Ambuffy* having an anti-apoptotic role also in this ovarian region. The high expression of *Ambuffy* in the basal stalk region seems to be contributing to the increase in transcript levels in the ovaries of workers at the PP phase, as detected by RT-qPCR.

In *Drosophila*, the function of Buffy may change dependently on specific cellular contexts. Buffy was first described as an anti-apoptotic protein [30], but was then seen to be necessary for promoting cell death in microchaete glial cells and in eye cells [55]. In addition, its over-expression promoted cell death in cultured cells [56]. This bifunctionality of Buffy has been observed not only in *Drosophila* but also in other organisms. There is evidence that in addition to regulating apoptosis, Bcl-2 family proteins have physiological functions as active components of cellular homeostatic pathways. For example, several Bcl-2 proteins regulate intracellular Ca^2+^ stores and the homeostatic autophagic pathway [57], [58]. As *Ambuffy* is the only Bcl-2 family member in *A. mellifera*, a function other than inhibition of cell death is plausible and should be considered in future studies.

In summary, we could establish a relationship between morphological events leading to caste-specific ovary differentiation in the honeybee and the expression of two conserved cell death genes. Consistent with a role as a cell death activator, *Amark* was upregulated in the ovarioles of workers, but not of queens, at the end of the fifth larval instar. *Amark* transcripts were found mainly in the apical ends of the worker ovarioles that die in consequence of the caste differentiation program. During the same developmental phases, *Ambuffy* expression increased earlier in the ovaries of queens than workers, and was localized in all queen ovarioles, but only in part of the worker ovarioles, supposedly protecting them from cell death. These results contribute to the knowledge on caste-related developmental plasticity in a social insect model system.

## Material and Methods

### Honeybee Rearing and Ovary Collection

Queen and worker larvae of Africanized honeybees, *A. mellifera*, were collected from hives of the Experimental Apiary of the Department of Genetics, University of São Paulo, Ribeirão Preto, Brazil. Standard apicultural techniques were used to rear queens by grafting first instar female larvae to queen cells. Worker and queen larval instars were determined using morphological criteria [59], [60]. Ovaries were dissected in cold Ringer saline, and processed for transcript quantification (RT-qPCR) or for spatial transcript localization (FISH). Digital images were obtained using a PlanS 1,0x, FWD 81 mm objective in a Discovery.V12 Stereomicroscope (Carl Zeiss MicroImaging GmbH, Jena, Germany) with an AxioCam MRc5 camera system. The developmental phases and the respective ovaries used in this study are shown in Figure 2.

### Identification of *Amark* and *Ambuffy* Genes

*Amark* and *Ambuffy* cell death genes were identified using Ark (isoforms A and B) (GenBank accession numbers AAM68488 and AAF57916) and Buffy (GenBank accession number AAF58628) protein sequences from *D. melanogaster* (FlyBase, http://flybase.bio.indiana.edu) as queries in BLASTP and TBLASTN searches against the honeybee Official Gene Set (OGS) v3.2 (http://www.hgsc.bcm.tmc.edu/projects/honeybee/). The mutual best BLAST hits were aligned, and these gene sequences were mapped against the honey bee genome using Artemis 7.0 software [61] (implemented in a LINUX server) in order to confirm gene identity and for intron/exon boundaries identification. The presence of conserved domains in the predicted proteins was checked by the following bioinformatics tools: SMART [62] and Conserved Domain Database [63].

The nucleotide sequences of *Amark* and *Ambuffy* were used as templates to design specific primers (Table S1) for PCR amplification of the first-strand cDNAs obtained by reverse transcription from total RNA extracted from worker and queen ovaries. Amplicons were purified and subcloned using TOPO TA-cloning kit (Invitrogen, Life Technologies Corporation, USA). Insert-containing plasmids were sequenced using specific- or M13 universal primers and ABI Prism BigDye Terminator Cycle Sequencing reagents (Applied Biosystems, Life Technologies Corporation, USA) in an automated sequencer ABI PRISM 310 Genetic Analyser (Applied Biosystems, Life Technologies Corporation, USA). Sequences were analyzed using Sequencher (version 4.7, Gene Codes Corporation) and the consensus sequence for each gene was aligned with the latest version (Amel 4.5) of the honeybee genome (Official Gene Set v.3.2) (Text S1 and Text S2).

### RT-qPCR

The expression of *Amark* and *Ambuffy* genes in the ovaries was accessed by RT-qPCR performed in a 7500 Real Time PCR system (Applied Biosystems). Primers were designed to amplify cDNA fragments of 151 and 153 bp of the *Amark* and *Ambuffy* genes, respectively. The gene encoding the RP49 ribosomal protein (now renamed as RPL32) in the honeybee (GenBank accession number NM_001011587), which is expressed in similar levels during development [64], and was previously validated by us in larval ovaries (data not shown), was used as reference. Each sample consisted of total RNA extracted from five ovary pairs using Trizol reagent (Invitrogen). RNA purity and concentrations were determined spectrophotometrically by means of a Nanodrop-1000 system (Thermo Scientific, USA). To remove remnants of genomic DNA, the RNA samples were incubated at 37°C in the presence of 1 U of RQ RNAse-free DNAse (Promega, USA) for 40 min, followed by 15 min at 70°C to inactivate the enzyme. First strand cDNA was synthesized from 1 µg of total RNA using SuperScript II reverse transcriptase (Invitrogen) and an Oligo(dT)_12–18_ primer (Invitrogen). PCR reactions were performed in a reaction mix containing 1×SYBR Green (Applied Biosystems, Life Technologies, USA), 10 pmol of each primer (Table S1) and 1 µL of first-strand cDNA in a final reaction volume of 20 µL. Amplifications were made under the following conditions: an initial cycle of 50°C for 2 min, a denaturation step of 95°C for 10 min and a two-step cycling condition (40 cycles of 95°C for 15 s and 60°C for 1 min). Ovaries of each developmental stage were assayed using three independent cDNA samples, each composed of five ovary pairs. Reactions were done in technical triplicates to check reproducibility. Baseline and threshold were set to obtain accurate CT values, which were then used for relative quantification of transcripts by the 2^−ΔΔCT^ method [65]. The data were analyzed by one-way ANOVA with *post-hoc* comparisons by the Holm-Sidak test using SigmaStat 3.1 software (Jandel Corporation, San Rafael, CA, USA), considering p<0.05 as statistically significant.

### FISH

FISH assays were carried out to localize *Amark* and *Ambuffy* transcripts in the ovaries. Primers were designed to amplify specific fragments of *Amark* and *Ambuffy* sequences (Table S1). Single-stranded antisense and sense probes were synthesized using the *FISH Tag RNA Green kit* or *FISH Tag RNA Orange kit* following manufacturer’s (Invitrogen) instructions.

Ovary fixation and processing steps were adapted from the protocol described for whole-mount ovaries of *Drosophila* [66]. Hybridizations were performed at least twice for all developmental stages and for both castes. Individual samples were composed of five to ten ovary pairs dissected in cold Ringer saline and fixed in heptane fixative [1 mL heptane, 80 µL HEPES buffer (0.1 M HEPES, pH 6.9, 2 mM MgSO4, 1 mM EGTA), 100 µL 8% paraformaldehyde, 20 µL dimethyl sulfoxide (DMSO)] for 30 min under shaking. The samples were quickly rinsed in absolute methanol (two rinses) and in absolute ethanol (two rinses), and then stored at −20°C, or immediately rehydrated in phosphate buffered saline (PBS) pH 7.4 containing 0.1% Tween-20 (PTw). After additional fixation during 20 min in a mixture of fixative (4% paraformaldehyde and 0.1% Triton X-100 in PBS and DMSO (9∶1 v/v), the samples were washed in PTw. To facilitate permeabilization and mRNA probe penetration, the samples were incubated for 5 min in a freshly prepared solution of 20 µg/mL proteinase K in PTw, followed by washes in a filter-sterilized solution of 10 mg glycine in 1 mL PTw. The ovaries were then rinsed in PTw and re-fixed, as above. After repeated washes in PTw, the samples were equilibrated in hybridization solution (HS), first in 1∶1 PTw/HS and subsequently in HS, this consisting of 50% formamide, 4x standard saline citrate, 1x Denhardt’s solution, 250 µg/mL yeast total RNA, 250 µg/mL boiled DNA from salmon testes, 50 µg/mL heparin, 0.1% Tween 20 and 5% dextran sulfate. Pre-hybridization in HS was done for 1h at 45°C. Sense and antisense probes were separately diluted in HS (200 ng/mL), heat-denatured for 2 min at 80°C, chilled on ice and added to the pre-hybridized samples. Hybridization was carried out overnight at 45°C under gentle shaking. The hybridized samples were washed in HS and PTw (3∶1, 1∶1 and 1∶3 v/v), and subsequently in PTw solution. For cell nuclei localization, the samples were post-stained with diamidino-2-phenylindole (DAPI) (1∶4000 in PTw), and washed in PTw. The ovaries were transferred to 70% glycerol in PTw, and mounted on slides using SlowFade Gold (Invitrogen) for observation under a Leica TCS-SP5 or TCS-SP2 Laser Scanning Confocal Microscope (LSCM; Leica, Germany).

## Supporting Information

Figure S1 ***Amark*** **gene expression in the ovaries of workers (W) and queens (Q) at the fourth larval instar (L4), at the feeding phase of the fifth larval instar (L5F) and at the last phase of the fifth larval instar (PP).**
(DOCX)

Table S1 **Primers used in qPCR analysis and fluorescence** ***in situ*** **hybridization for** ***Amark*** **and** ***Ambuffy*** **genes.**
(DOC)

Text S1 ***Amark*** **sequence alignment.** ClustalW alignment of predicted *Amark* sequence.
(DOC)

Text S2 ***Ambuffy*** **sequence alignment.** ClustalW alignment of the predicted *Ambuffy* 953 sequence.
(DOC)
