# Supplementary material for: Dimorphic Ovary Differentiation in Honeybee (Apis mellifera) Larvae Involves Caste-Specific Expression of Homologs of Ark and Buffy Cell Death Genes
Source: PLoS One. 2014 May 20;9(5):e98088. doi: 10.1371/journal.pone.0098088 (PMC4028266; doi:10.1371/journal.pone.0098088)

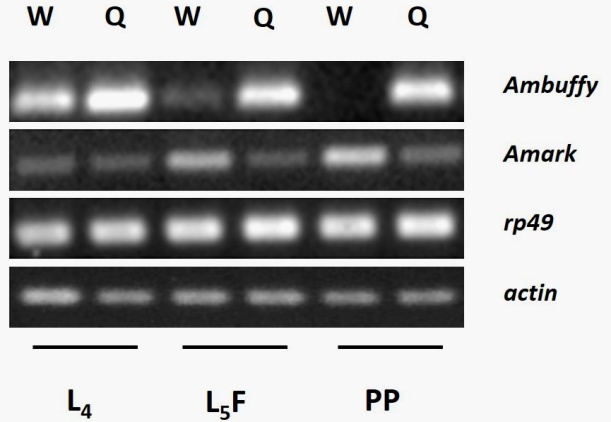


**Supporting Information Figure S1 -** *Amark* gene expression in the ovaries of workers (W) and queens (Q) at the fourth larval instar (L_4_), at the feeding phase of the fifth larval instar (L_5_F) and at the prepupal phase (PP). Semiquantitative RT-PCR using *rp49* (GenBank accession number NM_001011587) and *actin* (GenBank accession numberAB023025.1) as reference genes. Each sample is a pool made with five ovary pairs. PCR products were detected after electrophoresis on ethidium bromide-stained agarose gel.


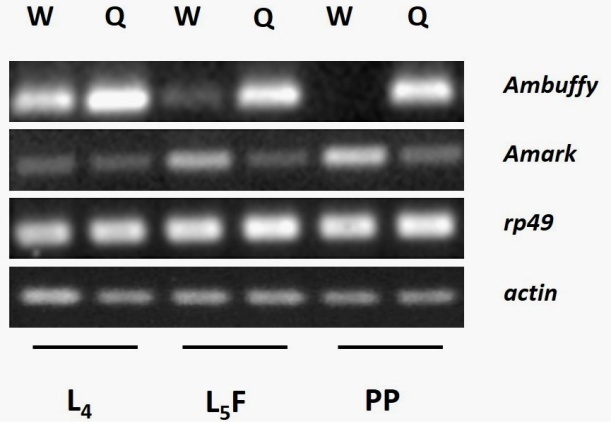

Supplement: Figure S1 — Amark gene expression in the ovaries of workers (W) and queens (Q) at the fourth larval instar (L4), at the feeding phase of the fifth larval instar (L5F) and at the last phase of the fifth larval instar (PP). (DOCX) [file pone.0098088.s001.docx]
