# Supplementary material for: Dimorphic Ovary Differentiation in Honeybee (Apis mellifera) Larvae Involves Caste-Specific Expression of Homologs of Ark and Buffy Cell Death Genes
Source: PLoS One. 2014 May 20;9(5):e98088. doi: 10.1371/journal.pone.0098088 (PMC4028266; doi:10.1371/journal.pone.0098088)
Supplement: Table S1 — Primers used in qPCR analysis and fluorescence in situ hybridization for Amark and Ambuffy genes. (DOC) [file pone.0098088.s002.doc]

**Table S1. Primers used in qPCR analysis and fluorescence *in situ* hybridization for *Amark* and *Ambuffy* genes**

| **Gene primer** | **Sequence 5’- 3’** | **Purpose** |
| --- | --- | --- |
| Amark - F | GTTTGTGCCAGTATGACTGA | Real Time PCR, *in situ* hybridization and sequencing |
| Amark - R | CCAATATGTGTCCAAAGAAGAA | Real Time PCR and sequencing |
| Amark - KoV - F | CCCAGAATCATTAAAGCCAGA | Real Time PCR |
| Amark - KoV - R | TTTGCAGCCAAAATCAAATG | Real Time PCR |
| Amark ISH - R | TGAACGCGTTTCCATAACAA | *In situ* hybridization and sequencing |
| Amark 1 - F | ATAAATTACATCAACATATTC | Sequencing |
| Amark 1 - R | ACGAGTGCTCGAATTATAAGG | Sequencing |
| Amark 2 - F | TCTCGTTTGTAGAGGGACAGA | Sequencing |
| Amark 2 - R | TCTCGTTTGTAGAGGGACAGA | Sequencing |
| Amark 3 - F | CGGCCTATTTCAGGTTGAAG | Sequencing |
| Amark 3 - R | ATTCCGCTCCAAAAAGATTG | Sequencing |
| Amark 4 - R | CGACAATAGCTAGTATTCCTG | Sequencing |
| Amark ISH T7 – F* | TAATACGACTCACTATAGGGCGAGTTTGTGCCAGTATGACTGA | *In situ* hybridization and sequencing |
| Amark ISH T7 – R* | TAATACGACTCACTATAGGGCGATGAACGCGTTTCCATAACAA | *In situ* hybridization and sequencing |
| Ambuffy Bcl – F | GGTATTGCCGTGGATTGTGT | Real Time PCR and sequencing |
| Ambuffy Bcl – R | CAGATCTGTATCGAGTTGCTA | Real Time PCR, *in situ* hybridization and sequencing |
| Ambuffy KoV – F | GGATCTTTACGTGGCTTGGA | Real Time PCR and sequencing |
| Ambuffy KoV – R | ATGTAGTTGATTCGGCTCGG | Real Time PCR and sequencing |
| Ambuffy Bcl HI – F | GGTGCAGTTGTGGGAGAAGT | *In situ* hybridization and sequencing |
| Ambuffy ISH T7 – F* | TAATACGACTCACTATAGGGCGAGGTGCAGTTGTGGGAGAAGT | *In situ* hybridization and sequencing |
| Ambuffy ISH T7 – R* | TAATACGACTCACTATAGGGCGACAGATCTGTATCGAGTTGCTA | *In situ* hybridization and sequencing |

The T7-promotor sequence is underlined in the primers used for probe template amplification on *In Situ Hybridization* experiments
