# Supplementary material for: Dimorphic Ovary Differentiation in Honeybee (Apis mellifera) Larvae Involves Caste-Specific Expression of Homologs of Ark and Buffy Cell Death Genes
Source: PLoS One. 2014 May 20;9(5):e98088. doi: 10.1371/journal.pone.0098088 (PMC4028266; doi:10.1371/journal.pone.0098088)
Supplement: Text S1 — Amark sequence alignment. ClustalW alignment of predicted Amark sequence. (DOC) [file pone.0098088.s003.doc]

**Text S1 – *Amark* sequence alignment.** ClustalW alignment of predicted *Amark* sequence (GB52453 - Official Gene Set v3.2) showing the consensus with the amplified fragments. The highlighted fragments correspond to conserved domains in the *Amark* coding region: Death Domain (red), NB-ARC (green) and WD repeats (blue).

GB52453 **ATGGATAAATTACATCAACATATTCTCAAAGTATTAAGAAAAAGTATAATGGCTGATATG**

**Sequenced -TGGATAAATTACATCAACATATTCTCAAAGTATTAAGAAAAAGTATAATGGCTGATATG**

***********************************************************

GB52453 **GATGTACATAATGGTATTATAAAGCTACTGCAATCAGAATATATATTAACAGATATACAA**

Sequenced **GATGTACACAATGGTATTATAAAGCTGCTGCAATCAGAATATATATTAACAGATATACAA**

******** ***************** *********************************

GB52453 **ATAGCAAGAATTGAAAATGGTGCTTCTAAAGAAGAAAAAGCTGAAATTTTATTAGATATA**

Sequenced **ATAGCAAGAATTGAAAATGGTGCTTCTAAAGAAGAAAAAGCTGAAATTTTATTAGATATA**

************************************************************

GB52453 **CTTCCAAGTTGTGGTCCAGGTGCATTTGATATTTTTCGTCAAGCTTTAAAACATCATTAT**

Sequenced **CTTCCAAGTTGTGGTCCAGGTGCATTTGATATTTTTCGTCAAGCTTTAAAACATCATTAT**

************************************************************

GB52453 **GAATGGTTAAGTAATGATATGGATAAACTTGAAGAA**AGTGTAAGATCATTTTGTGACATA

Sequenced **GAATGGTTAAGTAATGATATGGATAAACTTGAAGAA**AGTGTAAGATCATTTTGTGACATA

************************************************************

GB52453 AAGAATATCAGTACACCCACTCTTCCTCCAACATCACCATTAACTGTTGTA**AGAGAGGCA**

Sequenced AAGAATATCAGTACACCCACTCTTCCTCCAACATCACCATTAACTGTTGTA**AGAGAGGCA**

************************************************************

GB52453 **AAGATAAAACAGTTACAAAGTTATTTGGAAGAATTACAACCAAATGAATATATTGTACTT**

Sequenced **AAGATAAAACAGTTACAAAGTTATTTGGAAGAATTACAACCAAATGAATATATTGTACTT**

************************************************************

GB52453 **CATGGTATGAAAGGTTTTGGTAAATCATGTTTAACAGCTAGTACATTAAAAAACATGAAA**

Sequenced **CATGGTATGAAAGGTTTTGGTAAATCATGTTTAACAGCTAGTACATTAAAAAACATGAAA**

************************************************************

GB52453 **TTTGTAAGAAATATATTTTGTAATGAAGTGTATTGGATCAAATTTGGATATAAACGTCCA**

Sequenced **TTTGTAAGAAATATATTTTGTAATGAAGTGTATTGGATCAAATTTGGATATAAACGTCCA**

************************************************************

GB52453 **GTTGATGAAGAAATATTATTTCAACTTAATACATTGTATCATCGTGTCAAGAACTTGGAA**

Sequenced **GTTGATGAAGAAATATTATTTCAACTTAATACATTGTATCATCGTGTCAAGAACTTGGAA**

************************************************************

GB52453 **ATAGTCCCAGAATCATTAAAGCCAGAATCTTTAAAAGATTCATTAATTTATTTTTTGCAA**

Sequenced **ATAGTCCCAGAATCATTAAAGCCAGAATCTTTAAAAGATTCATTAATTTATTTTTTGCAA**

************************************************************

GB52453 **GAACATTTTAGTAGAGAAAAACATTGCCATGCTTTATTAATTTTAGATGATGTTTGTGAT**

Sequenced **GAACATTTTAGTAGAGAAAAACATTGCCATGCTTTATTAATTTTAGATGATGTTTGTGAT**

************************************************************

GB52453 **TCAAAAATAATTGAAACATTTGATTTTGGCTGCAAAACATTAGTAATTACAGCTGATCTT**

Sequenced **TCAAAAATAATTGAAACATTTGATTTTGGCTGCAAAACATTAGTAATTACAGCTGATCTT**

************************************************************

GB52453 **GATGTTGTATGGGAAAAAAGACCTCGTGTGATTGAGATGAATGATGGATTTACTGAAACA**

Sequenced **GATGTTGTATGGGAAAAAAGACCTCGTGTGATTGAGATGAATGATGGATTTACTGAAACA**

************************************************************

GB52453 **GAATCTTTAGAAGCAAAACGAATTCATGATGAATGTAAAGGAATGCCTTTATTAATTGCA**

Sequenced **GAATCTTTAGG**-------------------------------------------------

**********

GB52453 **ATGTTTTCTGCTCAATTTAAGGAATTTAAACATGATATGAAATTACGTCCAGATAGATGG**

Sequenced ------------------------------------------------------------

GB52453 **AAATACTATTTAAATTCATTAAGAAATAAGGATGAAAATAATCATGTAATTAAAAAATTT**

Sequenced ------------------------------------------------------------

GB52453 **TGGGAAAAACAAGAAGTAATTTTTAATATGTGTATAGAACAATTACCTCTTGAAATGAGA**

Sequenced ------------------------------------------------------------

GB52453 **AAACGTTATGAAGAATTAGTTATTTTTAGAGAAGATGTTAACATAACACCACAGACATTA**

Sequenced ------------------------------------------------------------

GB52453 GAAATTCTGTGGGAGGGCGGCCTATTTCAGGTTGAAGAAATGATGCTTGATTTATGTCAT

Sequenced ------------------------------------------------------TGTCAT

******

GB52453 AAGTCACTTGCGGCTAAACAATGGAATGACGAATTACGCAGTTATATATACGGTGTTCAT

Sequenced AAGTCACTTGCGGCTAAACAATGGAATGACGAATTACGCAGTTATATATACGGTGTTCAT

************************************************************

GB52453 GATTTATTACTTTGTCACCTTCGACAAAAATTTTCAAAAAATGAATTAATACAAATGCAT

Sequenced GATTTATTACTTTGTCACCTTCGACAAAAATTTTCAAAAAATGAATTAATACAAATGCAT

************************************************************

GB52453 AAATCATTGATTAAGAAATATCATAAATATTGCAATGGTGATTTTTCGAAATTACCAGCA

Sequenced AAATCATTGATTAAGAAATATCATAAATATTGCAATGGTGATTTTTCGAAATTACCAGCA

************************************************************

GB52453 GATAATTATAGTCATTCATATATTGGATATCATTTGGAGCAAGCAAAATTATATGATGAG

Sequenced GATAATTATAGTCATTCATATATTGGATATCATTTGGAGCAAGCAAAATTATATGATGAG

************************************************************

GB52453 TTTCCTAATATATATTTAGATTTTGATTTCATCCAACAAGCAATAGTCCATAGTGGTTTA

Sequenced TTTCCTAATATATATTTAGATTTTGATTTCATCCAACAAGCAATAGTCCATAGTGGTTTA

************************************************************

GB52453 AATAATTTATTGATTGATTTAAACAATTATAGAAAATATATTACCAAAGATTGTGATCCA

Sequenced AATAATTTATTGATTGATTTAAACAATTATAGAAAATATATTACCAAAGATTGTGATCCA

************************************************************

GB52453 GAATACGAAAAACGCTTTGTTGACTTGGAAAAATTTTTAGAAGAAAATTCAAGTACTATA

Sequenced GAATACGAAAAACGCTTTGTTGACTTGGAAAAATTTTTAGAAGAAAATTCAAGTACTATA

************************************************************

GB52453 GCAGAACATAGACGAAAAAAATGCTTGGATATAGTACAAATTGCTATGAGTCATCCTTAT

Sequenced GCAGAACATAGACGAAAAAAATGCTTGGATATAGTACAAATTGCTATGAGTCATCCTTAT

************************************************************

GB52453 CCGGGATACATTAAAGATACTGCTATTAAACTTGCAAAAGGAAGATCTAAATATTTATAT

Sequenced CCGGGATACATTAAAGATACTGCTATTAAACTTGCAAAAGGAAGATCTAAATATTTATAT

************************************************************

GB52453 TTATTTCATGATAA**AACAGGACAAATAGATATACCATTGAGTGAAGAAATGTCAACAGAA**

Sequenced TTATTTCATGATAA**AACAGGACAAATAGATATACCATTGAGTGAAGAAATGTCAACAGAA**

************************************************************

GB52453 **ATATGTACATCATGTTTTATAAATGATCCAAATCTTATATTAATTGGAAACCGATCGGGT**

Sequenced **ATATGTACATCATGTTTTATAAATGATCCAAATCTTATATTAATTGGAAACCGATCGGGT**

************************************************************

GB52453 **GAAATATTCTTATGGTAT**AGC**ATTTTCAAAAGGCAAAAAATTTTCAATGGACATGACAAA**

Sequenced **GAAATATTCTTATGGTAT**AGC**ATTTTCAAAAGGCAAAAAGTTTTCAATGGACATGACAAA**

*************************************** ********************

GB52453 **AATTCTTGCATAAAAAAAATTATTGTATGTAATGAAGGAGATTATTTCTTATCTTTAGAT**

Sequenced **AATTCTTGCATAAAAAAAATTATTGTATGTAATGAAGGAGATTATTTCTTATCTTTAGAT**

************************************************************

GB52453 **GATCGTGGTATAGTAAAATTATTTAAA**ATTTTTGATGATGAAAATTATGAAGAAAGTAAT

Sequenced **GATCGTGGTATAGTAAAATTATTTAAA**ATTTTTGATGATGAAAATTATGAAGAAAGTAAT

************************************************************

GB52453 AATGCTATATTAAGTCCAAGACAAAAACAATCTTTTTGGAGCGGAATTTTTACAAGTAAA

Sequenced AATGCTATATTAAGTCCAAGACAAAAACAATCTTTTTGGAGCGGAAT-------------

***********************************************

GB52453 ATTCC**TCGTGATGATAGTTCAGTAATATTTTCTGTTCCTAATGAAATTATATTAGATGTG**

Sequenced ------------------------------------------------------------

GB52453 **ACATTTTCACATGACAGTAGTTCTATTGCTGCTTGTACAAATAAAGGAACAATAAGGATT**

Sequenced ------------------------------------------------------------

GB52453 **TGGGAT**TGTCATGGAAATATATTATCAAATCACGGTCATAATCCTCAGAGTTCTCTTAAA

Sequenced -----------------------------------TCATAATCCTCAGAGTTCTCTTAAA

*************************

GB52453 AATATAGCATTTACATTAGAAAATAACATTACTTCTTTACTTCATATAATGGATGAAATA

Sequenced AATATAGCATTTACATTAGAAAATAACATTACTTCTTTACTTCATATAATGGATGAAATA

************************************************************

GB52453 CATGGTGTTATAATATCATATTGTAAATATGGTGATAAATATGAATATATATCAACATAC

Sequenced CATGGTGTTATAATATCATATTGTAAATATGGTGATAAATATGAATATATATCAACATAC

************************************************************

GB52453 AATTTAGATTTGAAAAAGAAGAAAGTTATCTTCTTCTGCAGTGTGCCACTACAAAATAAT

Sequenced AATTTAGATTTGAAAAAGAAGAAAGTTATCTTCTTCTGCAGTGTGCCACTACAAAATAAT

************************************************************

GB52453 TCTTTATTTATTGTTACTCAAAAAAAAGCTATATATGTAAAATGGTTTAGATCAAGTAAT

Sequenced TCTTTATTTATTGTTACTCAAAAAAAAGCTATATATGTAAAATGGTTTAGATCAAGTAAT

************************************************************

GB52453 AATCATATGCATAGTTA**TAATAAACAAGTAAGAGCAAGTGTTGAAAATGAAAAAACCGTT**

Sequenced AATCATATGCATAGTTA**TAATAAACAAGTAAGAGCAAGTGTTGAAAATGAAAAAACCGTT**

************************************************************

GB52453 **TATGTTTGTGCCAGTATGACTGATGATGGTCAGTATATAGTTTTAGCTGATTCTAGCGGT**

Sequenced **TATGTTTGTGCCAGTATGACTGATGATGGTCAGTATATAGTTTTAGCTGATTCTAGCGGT**

************************************************************

GB52453 **TTTATAAATGTATGGAAT**ATAAATAT**TGGACTTCATCCAATAACTACTTATAAAAGTCGT**

Sequenced **TTTATAAATGTATGGAAT**ATAAATAT**TGGACTTCATCCAATAACTACTTATAAAAGTCGT**

************************************************************

GB52453 **GTTTCTTCTTTGGACACATATTGGTTAAAAGATGAAGGGTATCATATTATATGTGGAAGT**

Sequenced **GTTTCTTCTTTGGACACATATTGGTTAAAAGATGAAGGGTATCATATTATATGTGGAAGT**

************************************************************

GB52453 **GAGAATCGATTACTTCATAAATGGAAA**TTTCCAGTAGAAGGAACTGGTATATCAATAAAA

Sequenced **GAGAATCGATTACTTCATAAATGGAAA**TTTCCAGTAGAAGGAACTGGTATATCAATAAAA

************************************************************

GB52453 AAACCTTTATTTGATGCAAAAGTACAGAATTTTGGCAATACTTCGGATACTATTGTTATG

Sequenced AAACCTTTATTTGATGCAAAAGTACAGAATTTTGGCAATACTTCGGATACTATTGTTATG

************************************************************

GB52453 GAAACGCGTTCAAATACTATTATTACATTAATTGGTGATGATAAAATAGCAGAAACTGAA

Sequenced GAAACGCGTTCAAATACTATTATTACATTAATTGGTGATGATAAAATAGCAGAAACTGAA

************************************************************

GB52453 CAAATTGATGGAAAAATAAATAATTTAATTCTTTATGCAGATAAAATAATTTATGTTACT

Sequenced CAAATTGATGGAAAAATAAATAATTTAATTCTTTATGCAGATAAAATAATTTATGTTACT

************************************************************

GB52453 GATAAAGTATTAGATATAAAAAAGGATATAATTAACAATGGTATTCTCGTTTGTAGAGGG

Sequenced GATAAAG-----------------------------------------------------

*******

GB52453 ACAGATGATAATTTACAGGTGTGGGA**AAACATAAAATTAAAATATATAATTCCGAATACG**

Sequenced ------------------------------------------------------------

GB52453 **GGTTACGTTATTGCAATTCATACAATTGATCAAGAATGTTTAGTAACAATAACTCGAAAT**

Sequenced ------------------------------------------------------------

GB52453 **GGTATAATAACAATTTGGAAT**ATCAAAAATATGAATTGGTTACAAACGGATAGAGTGAAC

Sequenced ------------------------------------------------------------

GB52453 GGAAATTCAGAAATAATCTTTAGTTGTTTAAGCTATCAGAAGAATTTCTTAGCTGTGTTA

Sequenced ------------------------------------------------------------

GB52453 AATGAAAATAGAGATGTGGTTTTATATAAATTACAGAAAGATATAATATTAAATCCAGCA

Sequenced ------------------------------------------------------------

GB52453 TATATTAAGATAAT**AGAATGTTCTAGGCTAACATATATTCATAAATTAACATGTTGTGAA**

Sequenced -------AGATAAT**AGAATGTTCTAGGCTAACATATATTCATAAATTAACATGTTGTGAA**

*****************************************************

GB52453 **ATTTCACAAAACGAAAAGTATTTAGCTATAGGTTTTGAAAATGGAAACATTTCTATTATT**

Sequenced **ATTTCACAAAACGAAAAGTATTTAGCTATAGGTTTTGAAAATGGAAACATTTCTATTATT**

************************************************************

GB52453 **GAT**ACATT**TACATTCGAAGAAATACGAAAATTGAACTTTCATACAAGTTCAGTTACTCAA**

Sequenced **GAT**ACATT**TACATTCGAAGAAATACGAAAATTGAACTTTCATACAAGTTCAGTTACTCAA**

************************************************************

GB52453 **TTACATTGGGCTCCCTCTATGATTGAAATTCCAATTTTGCTTTCTGTAAGTTCTGATGAA**

Sequenced **TTACATTGGGCTCCCTCTATGATTGAAATTCCAATTTTGCTTTCTGTAAGTTCTGATGAA**

************************************************************

GB52453 **TTAATTTGGTGGAAT**ATTACTTTAATTCTACACATACGTAAACCAAAATCGGAGAAAAGA

Sequenced **TTAATTTGGTGGAAT**ATTACTTTAATTCTACACATACGTAAACCAAAATCGGAGAAAAGA

************************************************************

GB52453 ACTGTATTGAATCACAGTTTTAGTTCTCCTTCTGTTAGTAAAATAACTAATGATTTCCCA

Sequenced ACTGTATTGAATCACAGTTTTAGTTCTCCTTCTGTTAGTAAAATAACTAATGATTTCCCA

************************************************************

GB52453 CATATATCTACTAGTCAAAGTATAGATTCGTATATTTGCCATTTACAAAATCAGCTACAA

Sequenced CATATATCTACTAGTCAAAGTATAGATTCGTATATTTGCCATTTACAAAATCAGCTACAA

************************************************************

GB52453 GGTAAAAATACAAAGAATGATATAGATAAACTAACTAAGTTTTGGAAATCTAAAGAGGGT

Sequenced GGTAAAAATACAAAGAATGATATAGATAAACTAACTAAGTTTTGGAAATCTAAAGAGGG-

***********************************************************

GB52453 AAAGATCCCAAACAACCAGGAATACTAGCTATTGTCGAATTACCTTCAAATTTTTTTGCT

Sequenced ------------------------------------------------------------

GB52453 AAAATATGCGTATCTACCGACTTCACTAAATATGTTACAGTTGATATATATGGATCAATC

Sequenced ------------------------------------------------------------

GB52453 AGTACTTTTACATTATGTGGGTATGATTAA---

Sequenced ---------------------------------
