# Supplementary material for: Dimorphic Ovary Differentiation in Honeybee (Apis mellifera) Larvae Involves Caste-Specific Expression of Homologs of Ark and Buffy Cell Death Genes
Source: PLoS One. 2014 May 20;9(5):e98088. doi: 10.1371/journal.pone.0098088 (PMC4028266; doi:10.1371/journal.pone.0098088)
Supplement: Text S2 — Ambuffy sequence alignment. ClustalW alignment of the predicted Ambuffy 953 sequence. (DOC) [file pone.0098088.s004.doc]

**Text S2 – *Ambuffy* sequence alignment.** ClustalW alignment of the predicted *Ambuffy* sequence (GB49154 - Official Gene Set v3.2) showing the consensus with the amplified fragments. The highlighted fragments correspond to conserved domains in the *Ambuffy* coding region: BCL Domain (pink) and Transmembrane Domain (orange).

GB49154 ATGAAGATGGATTTTCATTTGAGGGTGCAGGAGGAGGCGGCATTGCCGATGCCTGAAAAG

Sequenced ------------------------------------------------------------

GB49154 TCTGAATGGCAGGAATTCTCCACGGAAGATCCTTCAATGTCTGGATTCGTCGGATCTTTA

Sequenced ---------------------------------------------------GGATCTTTA

*********

GB49154 CGTGGCTTGGAGGACAATTCTGGGAATGCAGGCCAACCTTATCGCAGAAATAGTCTCGCA

Sequenced CGTGGCTTGGAGGACAATTCTGGGAATGCAGGCCAACCTTATCGCAGAAATAGTCTCGCA

************************************************************

GB49154 TTATCTCTTCATTCGAATTTGGCAGCTTTTCCTGTTCCTAATAATCAAGAAATCTCACCT

Sequenced TTATCTCTTCATTCGAATTTGGCAGCTTTTCCTGTTCCTAATAATCAAGAAATCTCACCT

************************************************************

GB49154 TTTCATGTAGTTGATTCGGCTCGGAGAAGATTTAGTAATGTCAGTGATGTCGTATCCAGA

Sequenced TTTCATGTAGTTGATTCGGCTCGGAGAAGATTTAGTAATGTCAGTGATGTCGTATCCAGA

************************************************************

GB49154 AAAATTTCTCATACAATTCGATGGAGAACGGTTTCAGCATCAATCGAGCTTACAGTATCT

Sequenced AAAATTTCTCACACAATTCGATGGAGAACGGTTTCG-CATCAATCGAGCTTACAGTATCT

*********** ***********************. ***********************

GB49154 CAAGGATCCTCCTTATGTGCT**CAATATATCCGAAATCGTTTGAAACGGTCTGGAATCTTT**

Sequenced CAAGGATCCTCCTTATGTGCT**CAATATATCCGAAATCGTTTGAAACGGTCTGGAATCTTT**

************************************************************

GB49154 **CATCGAAAGCTTGGATTGAAAAGGATGAGAAGTGCCATGTTGCTTCCTGGTGGTGCAGTT**

Sequenced **CATCGAAAGCTTGGATTGAAAAGGATGAG**-----------------**CTGGTGGTGCAGTT**

***************************** **************

GB49154 **GTGGGAGAAGTTTATCCGGAATTAGTATCAGTTGGAGCTGAACTCGAGAAAATGCATCCA**

Sequenced **GTGGGAGAAGTTTATCCGGAATTAGTATCAGTTGGAGCTGAACTCGAGAAAATGCATCCA**

************************************************************

GB49154 **AATTTATTCAATCGTGTTGCACGACAAATTGGATGTGGTAGTTTCTCATCGGAACAATCT**

Sequenced **AATTTATTCAATCGTGTTGCACGACAAATTGGATGTGGTAGTTTCTCATCGGAACAATCT**

************************************************************

GB49154 **GCTAGCGAGGCCATCGTGGATGTCTCTAGAGAGATGATCAGGAATGGTGAAATGACTTGG**

Sequenced **GCTAGCGAGGCCATCGTGGATGTCTCTAGAGAGATGATCAGGAATGGTGAAATGACTTGG**

************************************************************

GB49154 **AGTAAAGTGGTAGCCATTTATGCAATTGCTGGTGGTATTGCCGTGGATTGTGTACGTCAG**

Sequenced **AGTAAAGTGGTAGCCATTTATGCAATTGCTGGTGGTATTGCCGTGGATTGTGTACGTCAG**

************************************************************

GB49154 **GGCAAACCTGAATATTTACCTGCCATACAGAGAGGTATGACAGATGTTTTAGAAGAGGAT**

Sequenced **GGCAAACCTGAATATTTACCTGCCATACAGAGAGGTATGACAGATGTTTTAGAAGAGGAT**

************************************************************

GB49154 **CTTGCTGCATGGATCCAAGCTAACGGAGGATGGTCCGCTTTA**GCAACTCGATACAGATCT

Sequenced **CTTGCTGCATGGATCCAAGCTAACGGAGGATGGTCCGCTTTA**GCAACTCGATACAGATCT

************************************************************

GB49154 GTAACAAAAGAAACTACATGGCATTCGCGAAAACT**TATCTTGTTATTTATATTCACCATT**

Sequenced G-----------------------------------------------------------

*

GB49154 **TTGATTATTTTTATGATTTCTATGTTTTTAAAACTTTTAATT**TTA

Sequenced ---------------------------------------------
